# Dysregulated microRNA expression in rheumatoid arthritis families—a comparison between rheumatoid arthritis patients, their first-degree relatives, and healthy controls

**DOI:** 10.1007/s10067-020-05502-9

**Published:** 2020-11-18

**Authors:** Emma Renman, Mikael Brink, Lisbeth Ärlestig, Solbritt Rantapää-Dahlqvist, Kristina Lejon

**Affiliations:** 1 Department of Clinical Microbiology, Section of Infection and Immunology, Umeå University, SE-90185 Umeå, Sweden; 2 Department of Public Health and Clinical Medicine, Section of Medicine, Division of Rheumatology, Umeå University, SE-90185 Umeå, Sweden

**Keywords:** Autoimmunity, MicroRNA family, Rheumatoid arthritis, Sweden

## Abstract

**Objective:**

Recent studies have demonstrated an altered expression of certain microRNAs in patients with rheumatoid arthritis (RA) as well as their first-degree relatives (FDRs) compared to healthy controls (HCs), suggesting a role of microRNA in the progression of the disease. To corroborate this, a set of well-characterized RA families originating from northern Sweden were analyzed for differential expression of a selected set of microRNAs.

**Method:**

MicroRNA was isolated from frozen peripheral blood cells obtained from 21 different families and included 26 RA patients, 22 FDRs, and 21 HCs. Expression of the selected microRNAs miR-22-3p, miR-26b-5p, miR-34a-3p, miR-103a-3p, miR-142-3p, miR-146a-5p, miR-155, miR-346, and miR-451a was determined by a two-step quantitative real-time polymerase chain reaction (qRT-PCR). Statistical analysis including clinical variables was applied.

**Results:**

Out of the nine selected microRNAs that previously have been linked to RA, we confirmed four after adjusting for age and gender, i.e., miR-22-3p (*p* = 0.020), miR-26b-5p (*p* = 0.018), miR-142-3p (*p* = 0.005), and miR-155 (*p* = 0.033). Moreover, a significant trend with an intermediate microRNA expression in FDR was observed for the same four microRNAs. In addition, analysis of the effect of corticosteroid use showed modulation of miR-103a-3p expression.

**Conclusions:**

We confirm that microRNAs seem to be involved in the development of RA, and that the expression pattern in FDR is partly overlapping with RA patients. The contribution of single microRNAs in relation to the complex network including all microRNAs and other molecules is still to be revealed.

**Table:** 

**Key Points** *• Expression levels of miR-22-3p, miR-26b-5p, miR-142-3p, and miR-155 were significantly altered in RA patients compared to those in controls.* *• In first-degree relatives, a significant trend with an intermediate microRNA expression in FDR was observed for the same four microRNAs.*

## Introduction

Rheumatoid arthritis (RA) is a chronic autoimmune disease that affects 0.3–1% of the world population. RA is more common in northern Europe and North America, with predominance among women and elderly [1]. Previous studies suggest an interplay between genetic factors, environmental factors, and the immune system [2–4] although the pathogenesis of RA is still not fully understood. In families containing RA individuals, the majority of first-degree relatives (FDRs) never develop RA, even if they display risk factors such as smoking, anti-citrullinated protein antibodies (ACPAs), or carriage of HLA-shared epitope.

MicroRNA (miRNA or miR) is a non-coding sequence of single-stranded RNA that is approximately 18–25 nucleotides in length [5]. Its main function is to alter gene expression by post-transcriptional modifications and has shown to regulate about 30% of the protein coding genes. The mature microRNA will bind to a complementary sequence on mRNA that, depending on the grade of complementarity, will either lead to degradation of the mRNA or interfere with the translation process. Regarding this, microRNA primarily downregulates gene expression [2, 6]. A single microRNA can have the ability to regulate multiple genes, and it is also possible that one gene can be regulated by several microRNAs [2].

In a recent study based on indigenous North Americans, Anaparti et al. have shown an altered expression of several microRNAs in blood samples of RA patients as well as their FDRs, compared to healthy controls (HCs) [5]. Several other studies have similarly identified a dysregulated expression of microRNAs within the inflamed joints [7–10] and serum [11] as well as peripheral blood mononuclear cells (PBMCs) [12] from patients with RA, indicating that microRNA might have a role in the pathogenesis of the disease.

In a previous study of RA patients, their FDRs, and HCs originating from the four northernmost counties of Sweden [13], we have shown differences in the levels of IgG, IgA, and IgM ACPAs as well as of rheumatoid factor (RF) of IgM and IgA isotype. However, microRNA expression in  peripheral blood cells has not been analyzed in this group. In this study, the expression of a number of carefully chosen microRNAs was studied. Thus, miR-22-3p, miR-26b-5p, miR-34a-3p, miR-103a-3p, miR-142-3p, miR-146a-5p, miR-155, miR-346, and miR-451a were examined in peripheral blood cells of our cohort comparing the three groups: patients with RA, their FDRs, and HCs.

## Materials and methods

### Study design

Participants within this study originate from the four most northern counties in Sweden. The RA families were identified by a questionnaire provided to patients with RA at their respective rheumatology clinic. A total of 194 patients with confirmed diagnosis of RA (1987 ARA criteria) [14] and 191 unaffected FDRs were recruited from northern Sweden. All FDRs were interviewed by a questionnaire and all of the relatives with symptoms or signs of joint disease were clinically assessed by a rheumatologist as previously reported [13]. For this study, members from the 21 families living closest to the University Hospital in Umeå participated, resulting in 26 patients with RA and 22 FDRs. For comparison, we included 21 HCs in the study (Table 1). The FDRs included in the study were given a second questionnaire about symptoms and signs of joint disease and were clinically evaluated by a rheumatologist if signs of joint disease were reported. Blood samples were collected in PAXgene Blood RNA tubes (PreAnalytiX, Hombrechtikon, Switzerland) and were kept in − 80 °C until microRNA isolation.

**Table 1:** Descriptive characteristics of participants included in the study

	RA patients, *N* = 26	FDRs, *N* = 22	HCs, *N* = 21
Women/men	20/6	9/13	18/3
Age at sampling, mean (range), years	62.0 (42.7–83.0)	52.6 (31.3–79.6)	52.7 (28.3–71.4)
Anti-CCP positive, *n* (%)	24 (92.3)	8 (36.4)	ND
RF positive, *n* (%)	18 (69.2)	4 (18.2)	ND
Smoking, ever, *n* (%)	16 (61.5)	11 (50)	9 (42.9)
RA duration, mean (range), years	21.5 (5.8–51.0)	-	-
Treatment			
Corticosteroids, *n* (%)	7 (26.9)	1 (4.6)	-
sDMARD, *n* (%)	18 (69.2)	0	-
bDMARD, *n* (%)	4 (15.4)	0	-
NSAID, *n* (%)	20 (76.9)	2 (9.1)	-
Statins, *n* (%)	9 (34.6)	3 (13.6)	-

*ND* not determined

### Ethical considerations

All procedures performed in studies involving human participants were in accordance with the ethical standards of the institutional and/or national research committee and with the 1964 Helsinki declaration and its later amendments or comparable ethical standards. Informed consent was obtained from all individual participants included in the study. Ethical permits for this study were from the Regional Ethics Committee, Umeå (Dnr 05-068M and Dnr 2016-216-32M).

### Isolation of microRNA

The collected blood samples were defrosted in room temperature for 2 h and turned eight to ten times, then centrifuged for 10 min at 3000*g* in 4 °C using a centrifuge with a swing-out rotor (Allegra® X-15R Centrifuge, Beckman Coulter). The supernatant was removed and 1 mL Gibco™ PBS (pH 7.4, 1×, Thermo Fisher scientific, Waltham, MA, USA) was added to the pellet. The samples were transferred into 2-mL tubes and centrifuged for 10 min at 3000*g* in 4 °C and the supernatant was removed. The microRNA was isolated from the pellet using MirVana miRNA Isolation Kit (Life Technologies Europe B.V., Bleiswijk, Netherlands) according to the manufacturer’s instructions with the following changes: instead of acid-phenol:chloroform:isoamyl alcohol (125:24:1, pH 4.5 ± 0.2), phenol:chloroform:isoamyl alcohol (25:24:1, pH 6.7 ± 0.2) or phenol:chloroform:isoamyl alcohol (125:24:1, pH 4–5) with an additional volume of chloroform in a 2:1 ratio was used. Nuclease-free water was used as elution solution. The concentration of the isolated RNA was determined by a NanoDrop® ND-1000 Spectrophotometer with software NanoDrop 1000 3.7.1, or Thermo Scientific™ NanoDrop™ One C, where the absorbance at 230 nm, 260 nm, and 280 nm was measured. The samples were diluted with nuclease-free water to a final concentration of 2.0 ng/μL.

### Reverse transcription PCR

TaqMan® MicroRNA Reverse Transcription Kit (Life Technologies Europe B.V.) was used in order to perform the reverse transcription. RT primers from TaqMan® MicroRNA assays for RNU48 (assay ID: 001006), U6 snRNA (assay ID: 001973), hsa-miR-22-3p (assay ID: 000398), hsa-miR-26b-5p (assay ID: 000407), hsa-miR-34a-3p (assay ID: 002316), hsa-miR-103a-3p (assay ID: 000439), hsa-miR-142-3p (assay ID: 000464), hsa-miR-146a-5p (assay ID: 000468), hsa-miR-155 (assay ID: 002623), hsa-miR-346 (assay ID: 000553), and mmu-miR-451a (assay ID: 001141) with a concentration of 5× were used (Life Technologies Europe B.V.). The reverse transcription reaction was performed according to the protocol of the manufacturer for TaqMan® Small RNA Assays. The cDNA was amplified in PTC-100™ Programmable Thermal Controller, and the cDNA was stored in − 20 °C until qPCR was performed.

### Quantitative PCR

qPCR was performed using TaqMan™ Fast Universal PCR Master Mix (2×) no AmpErase™ UNG (Life Technologies Europe B.V.) and primers and probes from TaqMan® MicroRNA assays as mentioned above, with a concentration of 20× (Life Technologies Europe B.V.). The PCR reaction mix was prepared according to the manufacturer’s protocol for TaqMan® Small RNA Assays. Negative controls, without cDNA, were run on every plate. In the majority of cases, samples and negative controls were analyzed in triplicates. The qPCR was performed in QuantStudio5 and analyzed in QuantStudio™ Design and Analysis Software (Applied Biosystems, Foster City, CA, USA). For each of the nine microRNAs analyzed, the threshold value was adjusted to lay within the linear phase of the amplification curve and was held constant for all analyses of one particular microRNA. In initial analyses, two reference genes, i.e., RNU48 and U6 snRNA, were tested. The U6 snRNA was found to display the lowest deviation in expression in between the runs and was therefore selected as the reference gene.

The cycle threshold (*C*_T_) values received from the duplicates and triplicates were used to calculate a mean *C*_T_ value for each sample. An inter-experimental reference sample was included in all runs, and the mean *C*_T_ values for all microRNAs were adjusted to this reference sample. Δ*C*_T_ was calculated as [*C*_T_ (target) − *C*_T_ (U6 snRNA)] and used in order to calculate ΔΔ*C*_T_ [Δ*C*_T_ (RA) − Δ*C*_T_ (HC)], [Δ*C*_T_ (FDR) − Δ*C*_T_ (HC)], or [Δ*C*_T_ (RA) − Δ*C*_T_ (FDR)]. Fold difference in expression was calculated as 2^−ΔΔCT^.

### Statistical analysis and construction of figures

SPSS software version 26.0 (IBM, NY, USA) was used for statistical analyses. Comparisons between the groups of continuous variables were done using logistic regression presented with odds ratios (ORs) with 95% confidence intervals (CIs) or the Jonckheere-Terpstra test to analyze for the trend between the groups. Categorical variables were analyzed using the chi-square test. All tests were two-tailed. *p* values ≤ 0.05 were considered significant. The figures were created in GraphPad Prism 8 (version 8.4.3, GraphPad Software LLC, San Diego, CA, USA).

## Results

**Fig. 1:**
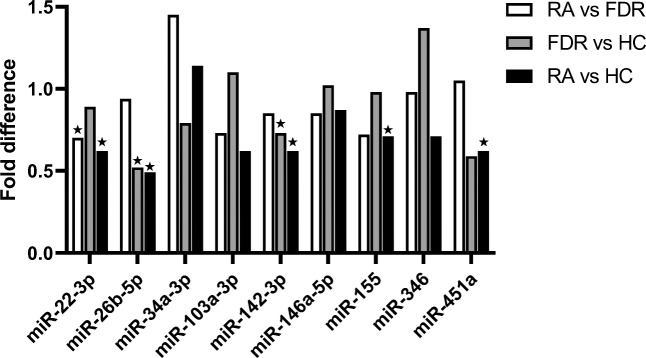
Graphical representation of observed fold differences between RA patients, FDRs, and HCs respectively for indicated microRNAs. A star indicates significant difference for the corresponding logistic regression analysis as described in Table 2

Analysis of the relative fold difference for the different microRNAs between the groups revealed that four of the nine microRNAs analyzed displayed significant differences in the comparison between RA and HCs (Table 2). In addition, difference in miR-155 expression was borderline significant in this comparison. After adjustments for age and gender, three microRNAs remained significant, i.e., miR-22-3p, miR-26b-5p, and miR-142-3p, and the *p* value of the miR-155 comparison became significant. Moreover, miR-26b-5p and miR-142-3p showed a significant difference comparing FDRs with HCs, where miR-26b-5p remained significant after adjustments. Notably, none of the analyzed microRNAs displayed significant difference between FDRs and RA after adjusting for gender and age (Table 2). A graphical overview of the findings is shown in Fig. 1.

**Table 2:** Fold difference in expression of microRNAs between RA patients, FDRs, and HCs (and *p* values from logistic regression)

		miR-22-3p	miR-26b-5p	miR-34a-3p	miR-103a-3p	miR-142-3p	miR-146a-5p	miR-155	miR-346	miR-451a
RA vs FDR	Fold difference	0.70	0.94	1.45	0.73	0.85	0.85	0.72	0.98	1.05
	OR^1^, 95% CI	0.317, 0.121–0.833	0.904, 0.548–1.492	1.277, 0.857–1.904	0.779 0.496–1.223	0.610 0.262–1.417	0.635, 0.282–1.432	0.547, 0.274–1.093	0.957, 0.466–1.963	0.949, 0.574–1.569
	*p* value	0.020*	0.694	0.229	0.277	0.250	0.274	0.088	0.904	0.838
	OR^2^, 95% CI	0.464, 0.163–1.325	1.028, 0.561–1.882	1.088, 0.689–1.718	0.834, 0.473–1.472	0.492, 0.181–1.335	0.677, 0.257–1.782	0.586, 0.263–1.305	0.928, 0.425–2.028	0.920, 0.500–1.694
	*p* value	0.152	0.929	0.718	0.532	0.164	0.430	0.191	0.851	0.789
FDR vs HC	Fold difference	0.89	0.52	0.79	1.10	0.73	1.02	0.98	1.37	0.59
	OR^1^, 95% CI	0.811, 0.419–1.571	0.565, 0.325–0.982	0.870, 0.590–1.284	1.176, 0.654–2.113	0.365, 0.137–0.975	1.073, 0.457–2.521	0.972, 0.511–1.850	1.661, 0.840–3.283	0.575, 0.328–1.009
	*p* value	0.534	0.043*	0.484	0.588	0.044*	0.871	0.931	0.145	0.054
	OR^2^, 95% CI	0.926, 0.460–1.862	0.521, 0.275–0.987	0.905, 0.583–1.404	1.200, 0.639–2.254	0.450, 0.150–1.351	1.144, 0.435–3.009	0.923, 0.434–1.960	1.888, 0.842–4.236	0.667, 0.366–1.216
	*p* value	0.828	0.045*	0.656	0.571	0.155	0.785	0.834	0.123	0.186
RA vs HC	Fold difference	0.62	0.49	1.14	0.80	0.62	0.87	0.71	1.35	0.62
	OR^1^, 95% CI	0.405, 0.191–0.859	0.425, 0.220–0.820	1.097, 0.737–1.632	0.860, 0.560–1.321	0.247, 0.095–0.644	0.661, 0.283–1.546	0.486, 0.229–1.031	1.987, 0.882–4.473	0.607, 0.372–0.988
	*p* value	0.019*	0.011*	0.648	0.491	0.004*	0.340	0.060	0.097	0.045*
	OR^2^, 95% CI	0.376, 0.165–0.855	0.384, 0.173–0.849	1.030, 0.677–1.568	0.853, 0.500–1.453	0.217, 0.75–0.625	0.631, 0.251–1.582	0.402, 0.174–0.931	1.489, 0.630–3.518	0.654, 0.389–1.098
	*p* value	0.020*	0.018*	0.889	0.557	0.005*	0.326	0.033*	0.364	0.108

Fold difference was calculated as 2^−ΔΔCT^ where HCs or FDRs are used as reference value. *p* values are obtained from logistic regression of microRNA values, OR^1^, 95% CI without adjustment (crude), and OR^2^, 95% CI with adjustment of age and gender. *Statistical significance, i.e., *p* ≤ 0.05

**Fig. 2:**
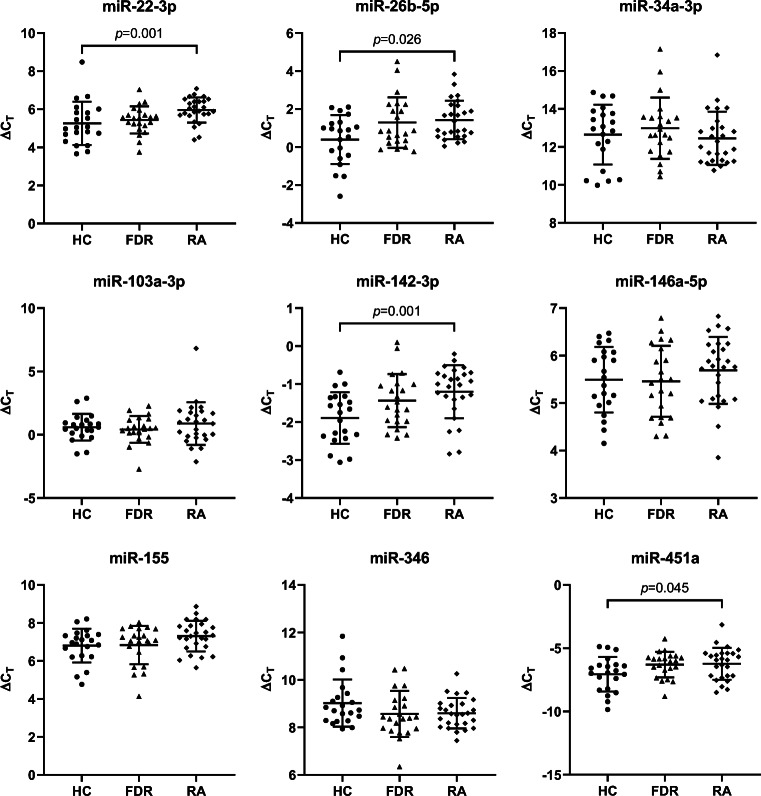
MicroRNA expression for RA patients, FDRs, and HCs. Δ*C*_T_ values for miR-22-3p, miR-26b-5p, miR-34a-3p, miR-103a-3p, miR-142-3p, miR-146a-5p, miR-155, miR-346, and miR-451a with mean values and standard deviation for RA patients, FDRs, and HCs noted out as solid lines. Expression of the miRNAs was adjusted to the reference gene U6 snRNA. The Jonckheere-Terpstra test was applied for each microRNA, where statistical significance between the three groups, *p* ≤ 0.05, is depicted in the figure

Next, we applied the Jonckheere-Terpstra test to compare the three groups regarding microRNA expression. We found significant differences between the three groups for miR-22-3p (*p* = 0.001), miR-26b-5p (*p* = 0.026), miR-142-3p (*p* = 0.001), and miR-451a (*p* = 0.045) as presented in Fig. 2. In addition, for miR-155, the difference between the three groups was borderline significant (*p* = 0.051). Principal component analysis based on relative expression level was applied to investigate potential clustering of individuals. No particular pattern was observed (data not shown).

Next, influence of treatments of the RA patients was determined, and a significant effect of corticosteroids on miR-103a-3p levels was observed, with an upregulation of miR-103a-3p in the group treated with corticosteroids (*p* = 0.048).

## Discussion

### miRNAs are involved in RA pathogenesis

Familial studies provide a unique possibility to evaluate the impact of potential contributing factors in the at-risk FDR group. Taking this approach, we have previously shown that the frequency of HLA-DRB1*0401/0404/0408 and the T variant of *PTPN22*, which are risk factors of RA development, are significantly increased in RA and FDRs compared to those in HCs [13]. Moreover, levels of ACPAs and RF in plasma, in particular of the IgA and IgM isotypes, were significantly different between the three groups, where FDRs showed an intermediate phenotype [13]. Despite the presence of risk factors in the FDRs for RA development, regulatory mechanisms appear to still be in place. MicroRNAs could potentially be involved in this process. Indeed, several recent studies [5, 11, 12, 15] have demonstrated both overlaps and differences in the expression of multiple microRNAs comparing RA patients, FDRs, and HCs.

Thus, in the current study, we have analyzed the expression levels of nine different microRNAs in peripheral blood cells of well-characterized RA, FDR, and HC individuals. Expression of miR-26b-5p and miR-142-3p was downregulated in FDRs compared to that in HCs, and miR-26b-5p remained significant after adjustment for age and gender. Comparison of FDR to RA patients showed that FDR and RA patients display similar levels of these microRNAs, suggesting that FDR display this disease-associated feature. Our observations are in line with Zhu et al. where downregulation of miR-26b-5p and miR-142-3p was observed in PBMC of RA patients compared with controls [12]. MiR-26b has been associated to RA and inflammation where inhibition of miR-26b in rheumatoid arthritis fibroblast-like synoviocytes (RAFLS) causes increased levels of TNF-α, IL-1β, and IL-6, while miR-26b mimics mediate downregulation of the same proinflammatory cytokines [16]. Chen et al. have found that miR-26 is able to downregulate IL-6 expression as well as TNF-α/NF-κB signaling, by targeting mucosa-associated lymphoid tissue lymphoma translocation protein 1 (MALT1) and high-mobility group AT-hook 1 (HMGA1) [17]. Moreover, levels of miR-26b are downregulated in the cartilage of osteoarthritis (OA) patients compared to those in controls [18], where decreased levels of miR-26b also have been suggested to contribute to disease progression by upregulation of the NF-κB signaling pathway. In our analysis, however, we did not observe a correlation between miR-26b and IL-6 or IL-1β in RA and FDR (data not shown), which indicates that regulation of the expression of these cytokines depends on multiple influencing factors. Our result also deviates from the study by Anaparti et al., where significant decreased levels of miR-26b-5p were observed in FDRs compared to RA, and when compared to HCs, no significant difference was noted [5].

In this study, miR-142-3p was shown to be significantly downregulated in RA (and FDRs before adjustment) compared to that in HCs, which is in line with the study by Zhu et al., comparing PBMCs in RA and HC [12]. However, in serum and RAFLS, miR-142-3p is upregulated in patients with very early RA and CCP-positive at-risk individuals [11], as well as in RA patients [19] compared to HCs. This corroborates the complexity of microRNA function and regulation including that miR-142-3p seems to play various roles in various tissues.

An extensively studied microRNA in the development of RA is miR-155, which has been found to be upregulated in RA patients and in FDRs compared to HCs or OA patients [5, 20, 21]. MiR-155 is associated with inflammation and has been associated with erythrocyte sedimentation rate (ESR), disease activity score (DAS-28), and plasma levels of proinflammatory cytokines [22] in RA patients. MiR-155 has several targets, including suppressor of cytokine signaling 1 (SOCS1) and SH-2 containing inositol 5′ polyphosphatase 1 (SHIP1). Inhibition of SOCS1 and SHIP1 by miR-155 results in an enhancement of inflammatory cytokine production, i.e., TNF, IL-1, and IL-6 [23], thus contributing to an inflammatory milieu. Other reported targets are the Fas-associated death domain (FADD) protein and the IκB kinase (IKK), which both are anti-inflammatory molecules [22]. This further supports the proinflammatory role of miR-155. Unexpectedly, in the current study, we found a decrease of miR-155 expression in RA patients compared to HCs after adjustment for age and gender, which deviate from previous findings. In addition, miR-146 that also has been associated with inflammation did not differ in our cohort. The underlying cause for this is still to be revealed, but one possibility is that the RA patients are undergoing treatments of various kinds and the disease was well controlled at the time of sampling.

This study found a significant downregulation of miR-22-3p in RA compared to both FDRs and HCs, and the significant difference remained between RA and HCs after adjustment for age and gender. Lin et al. found that miR-22 was downregulated in synovial tissue of RA patients compared to that of OA patients, and that the levels of miR-22 were negatively correlated to the levels of Cyr61 overexpression and the promotion of IL-6 production [24]. Similar results have been reported by Yu et al., where inhibition of p53 in subarachnoid hemorrhage (SAH) mice led to decreased levels of miR-22 and upregulation of Cyr61, whereas knock down of p53 in HEB cells (human normal glial cell line) suppressed the expression of miR-22 [25]. Decreased levels of miR-22 in synovial tissue of RA patients compared to HCs have also been reported and this is negatively correlated to sirtuin 1 (SIRT1) levels [26]. Interestingly, addition of a miR-22 mimic in this context leads to decreased levels of TNF-α, IL-1β, and IL-6, supporting the role of miR-22 in inflammation and RA pathogenesis [26].

The levels of miR-451a were in this study significantly decreased in RA compared to those in HCs, and borderline significant comparing FDRs and HCs (*p* = 0.054). However, after adjustment for age and gender, no significant difference remained, indicating that levels of miR-451a are highly influenced by these parameters. Meta-analysis of likely targets of miR-451a [27] includes macrophage migration inhibitory factor (MIF) that is negatively modulated by miR-451a [28] and IL-6R that is also negatively regulated by miR-451a [29]. As we observed a downregulation of miR-451a in RA patients compared to HC, we speculate that this could represent an underlying inflammation and thus upregulation of IL-6R and MIF.

In contrast to Anaparti et al. [5], we did not observe any upregulation of miR-103a-3p expression in RA patients and FDRs compared to HCs. However, we observed downregulation of miR-103a-3p in RA patients using corticosteroids, which suggests a modulating effect of this treatment in line with a beneficial effect of miR-103a-3p suppression. The role of miR-103a-3p in bone remodulation has been extensively studied in both animal models and in various rheumatoid diseases [30–32]. In addition, the influence of corticosteroids on both miR-103 and osteoporosis has demonstrated to be tightly connected [33–36].

When applying the Jonckheere-Terpstra test, four of the nine microRNAs analyzed displayed a significant trend comparing RA patients, FDRs, and HCs, where FDRs exhibited an intermediate phenotype. The intermediary pattern for the microRNAs in the FDRs resembles our previously published observations [13], where an intermediate level of ACPA and RF in FDRs of the same cohort was observed. Taken together, this suggests that a dysregulation of microRNAs may be involved in the increased risk of RA development in FDRs. However, for some microRNAs, the expression level did not differ when a group-wise comparison of FDRs and HCs was performed, suggesting that microRNAs could also have a protective role, preventing FDRs to develop the disease. Overall, regulation of inflammation and other biological mechanisms by microRNAs and other factors is highly complex, and cannot be expected to be explained by a single microRNA.

In summary, we confirm that microRNAs seem to play a role in promotion and protection of RA development. Strength of this study is the usage of a well-characterized cohort, as well as the familial analysis approach where the participants originate from a restricted geographic area, and display a genetic and environmental homogeneity.
